# Assessment of Farmers’ Knowledge, Attitudes and Practices on Antibiotics and Antimicrobial Resistance

**DOI:** 10.3390/ani9090653

**Published:** 2019-09-04

**Authors:** Yasin Ozturk, Senol Celik, Emre Sahin, Mehmet Nuri Acik, Burhan Cetinkaya

**Affiliations:** 1 Department of Pharmacology, Faculty of Veterinary Medicine, University of Bingol, 12000 Bingol, Turkey; 2 Department of Biostatistics, Faculty of Agriculture, University of Bingol, 12000 Bingol, Turkey; 3 Department of Animal Nutrition, Faculty of Veterinary Medicine, University of Bingol, 12000 Bingol, Turkey; 4 Department of Microbiology, Faculty of Veterinary Medicine, University of Bingol, 12000 Bingol, Turkey; 5 Department of Microbiology, Faculty of Veterinary Medicine, University of Firat, 23119 Elazig, Turkey

**Keywords:** antibiotic use, antibiotic knowledge level, antibiotic resistance, farmers, survey, questionnaire

## Abstract

**Simple Summary:**

Antibiotic resistance is a global problem that threatens human and animal health and has increased in recent years. Although many factors are responsible for the development of resistance, antibiotics used in animals for preventive, therapeutic, and other purposes play a major role. Conscious and rational antibiotic use in animals will contribute to decreases in resistance. It is therefore important to determine knowledge, attitudes, and behaviors of farmers working in the livestock sector with regard to antibiotic knowledge, use, and resistance in order to develop communication strategies accordingly. The aim of this study was to measure the knowledge of livestock farmers about antibiotics by conducting a questionnaire survey. As a result of the survey, it was found that knowledge of the participants on antibiotics and resistance was very low. It was concluded that periodic training programs can be employed to overcome this problem and raise awareness among farmers.

**Abstract:**

The aim of this study was to determine knowledge, attitudes, and behaviors of farmers dealing with animal husbandry in eastern Turkey with regard to antibiotic knowledge, use, and resistance. A face to face questionnaire survey, consisting of five sections with 42 questions in total, was applied to 360 farmers located in the region. The questions in the first and fifth sections were closed-ended while those in other sections were prepared using the Likert scale. It was determined that knowledge of the farmers about antibiotic use, duration, storage, and resistance was well below desired levels. This was particularly remarkable in the participants with a low level of education, living in rural areas, and those at 48 years of age or over. In contrast, younger and highly educated participants living in urban areas were more knowledgeable about antibiotic use and they were well aware of the fact that resistance might pose a great risk for public health. Providing appropriate antibiotic use in animals through systematic training of livestock farmers is crucial in tackling the resistance problem.

## 1. Introduction

The discovery of antibiotics commonly used in the treatment and control of infectious diseases caused by microorganisms has been an important milestone for human health. Many infections, especially epidemics that have been a major threat to human life in the past and have caused high mortality, have been brought under control following the use of antibiotics [1]. Similarly, antibiotics which reduce morbidity and mortality of infectious diseases significantly are the most commonly used chemicals for treatment and control of infections in animals. In addition, antibiotics are excessively used in animals, especially in ruminants, for the production of high-quality and low-cost food and for the purpose of raising healthier and more productive animals [2,3]. However, antibiotic resistance, which has rapidly increased in recent years and has become a global problem, makes it difficult to treat and control infections in both humans and animals. It is pointed out in the World Health Organization (WHO) reports that humanity is at risk of returning to the “pre-antibiotic era” and all available financial and scientific resources should be spent to prevent this risk [4,5]. In the same reports, it is underlined that even a small throat infection cause death and this problem of antimicrobial resistance can therefore be controlled only through the joint efforts of the countries knowing the fact that it is a social and global matter rather than individual and national [4,5]. Many factors, especially excessive and inappropriate use of antibiotics, play a role in the development of resistance. Failure to diagnose the disease correctly, patient’s demand, socio-cultural differences, and knowledge, belief, expectations, and attitudes of people toward antibiotics are also responsible for facilitating the emergence and spread of antibiotic-resistant microorganisms [6,7]. In addition, several misapplications by patients such as failure to complete treatment, skipping doses, re-use of residual drugs, misuse of antibiotics in the treatment of viral infections, and use of non-rational antibiotics, such as self-treatment, have been reported [8,9].

As in many countries, intensive efforts such as legal arrangements are being made in Turkey to combat antibiotic resistance. Although e-prescription and a drug tracking system for veterinary drugs was officially established in 2018, the system is not fully settled down due to the lack of adequate infrastructure. Veterinarians working both in public and private clinics continue to make prescriptions without entering the e-prescription system and animal owners continue to receive the drugs they wish from the clinics.

The increase in drug resistant bacterial infections in humans has led to studies on monitoring and controlling of all possibly responsible factors. In this context, animal products are another important factor with a role in resistance. Antimicrobial drugs are used extensively for the prevention, control, and treatment of diseases in animals [10,11]. Antibiotics used in animals can lead to low-level exposure through the consumption of animal products such as meat, milk, and eggs which contain drug residues. Zoonotic resistant bacteria (*Campylobacter*, *Escherichia coli*, *Salmonella*, *Enterococcus* spp., etc.) in the intestines of animals can be transferred to humans [11]. Therefore, it is very important to control the use of antibiotics, monitor resistance, and develop new strategies to reduce antimicrobial resistance in animals. One of the most important of these strategies is to increase knowledge and skills of the community about antibiotic use in order to raise awareness on this issue and influence behavior change. Veterinarians and farmers are the leading antibiotic users for the treatment and control of diseases in animals. It is possible to prevent intensive and unnecessary antibiotic use in animals by informing farmers about antibiotics, residues, and resistance which may decrease drug resistance in humans. However, there is a paucity of information on the knowledge, consciousness, and awareness of animal owners about antibiotics, residues, and resistance. It is very important to determine the current situation in the field in terms of establishing and assessing effective measures toward preventing antibiotic resistance. The current study was therefore carried out to evaluate knowledge, attitudes and practices of farmers in the east of Turkey on the use of antibiotics, residues, and resistance.

## 2. Material and Methods

### 2.1. Ethical Approval

Ethical approval was obtained from Scientific Research and Publication Ethics Committee, Bingol University. The consent of selected farmers were sought and obtained after explaining the purpose of the study (with protocol 11.07.2019-E.13913).

### 2.2. Survey Region

A questionnaire survey was carried out in cattle farms located in Bingol province and its central villages, in eastern Turkey, covering 1814 km^2^ surface area. Bingol province is suitable for cattle breeding due to its geographical structure and most of the local people live work in livestock production. In the study region, both beef and dairy cattle breeding are common and most of the animals are mixed breeds. Approximately 1% of the national cattle population of the country (n = 17 million) is present in Bingol. Most of the enterprises in the province are family type managements and the average number of animals in each enterprise is 12.

### 2.3. Sample Size

The sample size was calculated by using Raosoft sample volume calculation method based on a 5% error rate, 95% reliability level, and 50% response distribution [12]. The sample size was calculated as 360 in a total population of 5371 cattle breeders in Bingol and its central villages. The enterprises surveyed were selected by random sampling from 36 out of 78 locations which contain 20 or more cattle farms in Bingol and its central villages. Cattle breeders were also determined by random sampling with an equal number of participants from each of the identified locations (10 farms from each village).

### 2.4. Survey Method

Before conducting the survey in the field, a pilot study was carried out with 25 randomly selected animal owners who applied to the clinics of Bingol Veterinary Faculty in order to test the clarity, reliability, and validity of the questionnaire. Following this study, the questionnaire was revised and finalized in accordance with suggestions and data were collected in the study population. The questionnaire, which consisted of five sections and 42 questions, was constructed by taking into consideration the questions of the WHO organization and previous similar studies [5,13,14,15,16].

The first part of the questionnaire includes five questions concerning demographic information, the second part includes nine questions measuring general information about antibiotics, the third part includes 10 questions in relation to antibiotic use, the fourth part includes 10 (one closed-ended and nine five point Likert scale response options) questions with respect to antibiotic resistance, and the final part includes eight questions about duration of antibiotic use and storage. While the questions in the first and fifth sections were closed-ended, the questions in the second, third, and fourth sections were prepared using five point Likert scale response options. The questionnaire was applied to the farmers by the face to face survey method by trained and experienced researchers.

### 2.5. Data Analyses

The internal consistency of answers given to the questions which were prepared according to the 5-point Likert scale was measured with reliability analysis. The Cronbach’s α, which is a numerical coefficient of reliability, was determined as 0.68, 0.64, and 0.73, respectively for the questions asked in the second (antibiotic knowledge level), third (antibiotic use), and fourth (antibiotic resistance) sections.

The scores (totally agree = 1 point, agree = 2 points, neutral = 3 points, disagree = 4 points and totally disagree = 5 points) obtained from the answers given by the participants to the questions in the second, third, and fourth sections were collected and the section scores were calculated. The participant scores taken from each section were compared with one-way analysis of variance (ANOVA) for “educational status”, “age”, and “the time spent as farmer” while an independent *t* test was used for comparison of “place of residence”. The Levene test was performed to determine the homogeneity of variances. The results were presented as mean ± standard deviation (SD) and *p* < 0.05 was considered as statistically significant. Pearson correlation analysis was performed to determine the direction and degree of the relationship between the mean scores of the second, third, and fourth sections. IBM SPSS 22.0 for Windows (SPSS Inc., Chicago, IL, USA) was used for statistical analyses. GraphPad Prism for Windows version 5.0 (GraphPad software Inc., San Diego, CA, USA) was used to create charts. The stacked bar charts for Likert scales were created using Tableau 8.2 (Tableau Software, Washington, DC, USA).

## 3. Results

### 3.1. Demographic Information

Analysis of demographic parameters showed that the vast majority of the participants were male, primary school graduates, at the age of 48 years or more, and were engaged with animal breeding for at least 11 years. On the other hand, the percentages of female, university graduate, and younger (18–32 years old) cattle farmers were rather low (Table 1).

### 3.2. Antibiotic Knowledge Level

**Figure 1:**
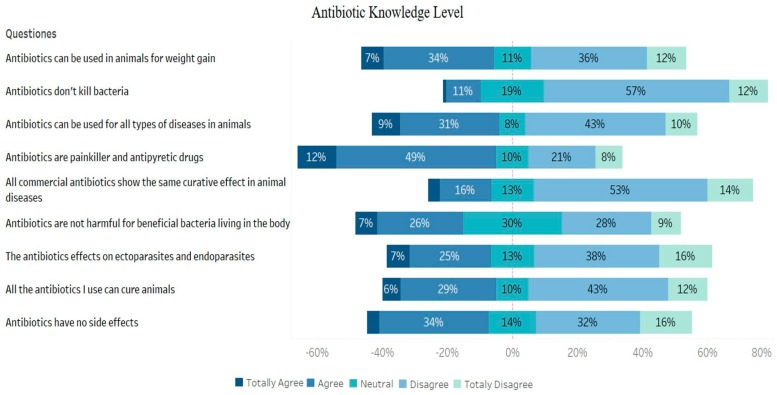
Findings of antibiotic knowledge level.

**Figure 2:**
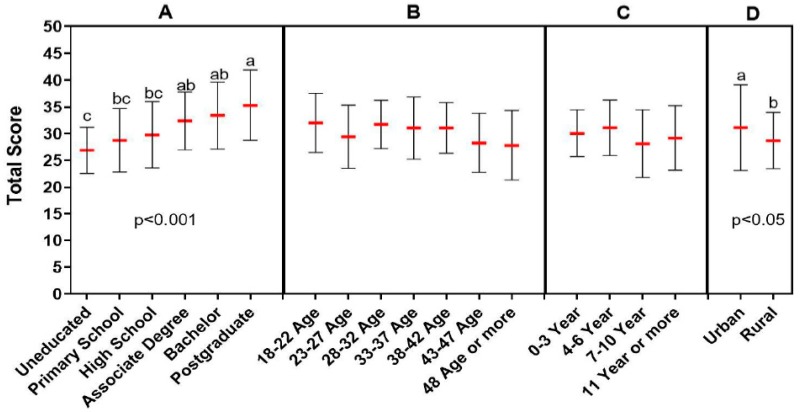
Association between antibiotic knowledge level and demographic data (**A**: educational status, **B**: age, **C**: the time spent as farmer, **D**: place of residence). Each demographic data group was analyzed independently. The red lines indicate the mean value and the error bars indicate the standard deviation. Lower case letters (a, b, c) above the groups indicate statistical difference between the groups that is *p* < 0.05 and *p* < 0.001.

It was found that a large proportion of the farmers participating in the survey had inadequate information about antibiotics and that they used antibiotics inappropriately. Approximately 38% of the farmers responded that antibiotics had no side effects, while the rate of those considering antibiotics as effective against parasites was as high as 32%. One of the interesting findings was that more than half of the participants considered antibiotics as antipyretic and analgesic drugs. Although antibiotic use for weight gain is prohibited in Turkey, approximately 41% of the participants stated that antibiotics could be used for this purpose in animals (Figure 1). A relationship was noted between education level and antibiotic knowledge level of the participants. Antibiotic knowledge level of the participants with a postgraduate degree was found to be significantly higher than high school and primary school graduates or uneducated (*p* < 0.001). Antibiotic knowledge of the participants with associate degrees and/or bachelor degrees was also significantly higher when compared to those without education (*p* < 0.001). Antibiotic knowledge level of the residents in urban areas was detected to be significantly higher than those living in rural areas (*p* < 0.05) (Figure 2).

### 3.3. Antibiotic Use

**Figure 3:**
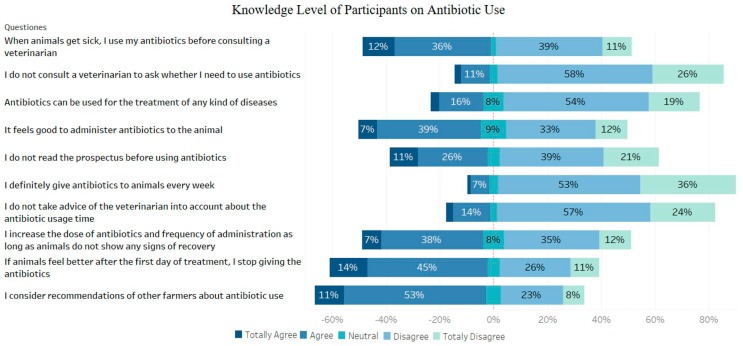
Knowledge level of participants on antibiotic use.

**Figure 4:**
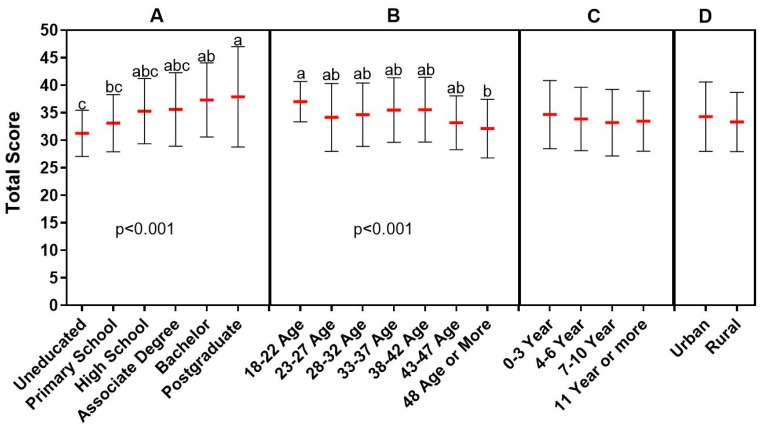
Relationship between knowledge level of participants on antibiotic use and demographic data. (**A**: educational status, **B**: age, **C**: the time spent as farmer, **D**: place of residence). Each demographic data group was analyzed independently. The red lines indicate the mean value and the error bars indicate the standard deviation. Lower case letters above the groups indicate statistical difference between the groups. Lower case letters (a,b,c) above the groups indicate statistical difference between the groups that is *p* < 0.001.

Approximately 64% of the respondents stated that they took advice from other farmers about antibiotic use and 48% did not need to consult a veterinarian before antibiotic use. While most of the rural farmers answered these questions positively, most of the farmers living in the city center stated that they did not agree with these views. Approximately 59% of the participants responded that they stopped giving the drug if animals recovered the day after the onset of antibiotics. On the other hand, 45% continued treatment with higher and more frequent doses as long as animals did not show any signs of recovery. Demographic factors such as age and educational level did not play a role on these questions as all the farmers participating in the survey gave similar answers. On the other hand, the vast majority of participants applied veterinarians’ opinions and recommendations on the need for antibiotics and read the label and package insert before using the antibiotics, which showed that the public was aware of and behaved sensitively in this respect (Figure 3). The knowledge level of the participants with postgraduate degree on antibiotic use was found to be significantly higher than those of the primary school graduate and uneducated (*p* < 0.001). Also, younger farmers (18–22 years old) had higher knowledge level on antibiotic use than older ones (48 years or older) (*p* < 0.001) (Figure 4).

### 3.4. Antibiotic Resistance

**Figure 5:**
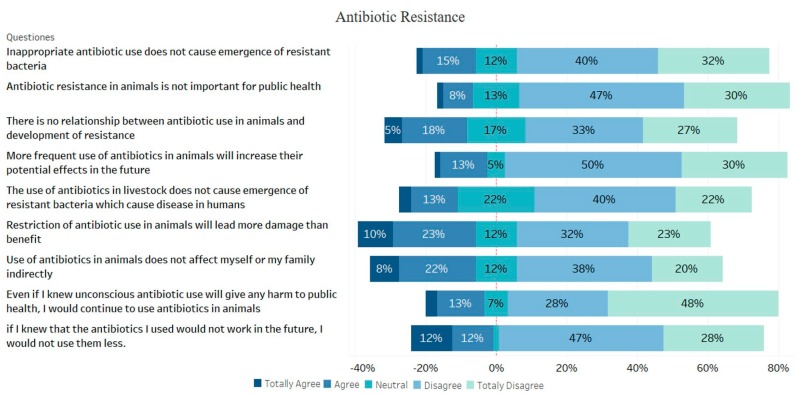
Findings obtained from the participant on antibiotic resistance.

**Figure 6:**
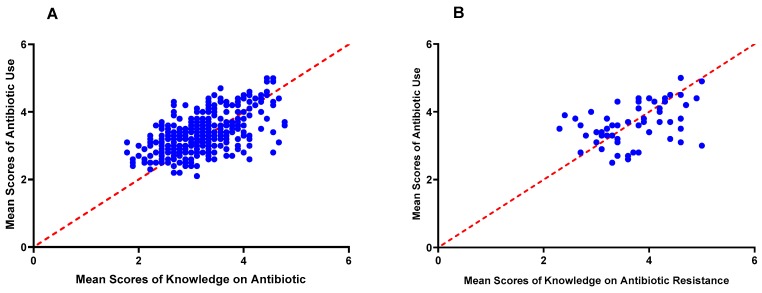
Correlation graphs showing the association between antibiotic use and knowledge on antibiotics (**A**) and knowledge on antibiotic resistance (**B**).

Before the five-choice questions about antibiotic resistance, the following question was asked to the participants: “Do you know about antibiotic resistance that has become a global problem?” Approximately 17% of the participants stated that they had information about the importance of antibiotic resistance. The questions listed in Figure 5 were then asked to the participants who had information about antibiotic resistance and 72% of them stated that inappropriate use of antibiotics caused the development of resistant bacteria and, and 77% were aware of the fact that antibiotic resistance was an important issue in terms of public health (Figure 3). The participants who were aware of the global antibiotic resistance problem had significantly higher levels than those who answered negatively to the question concerning importance of resistance in terms of antibiotic knowledge and appropriate antibiotic use (*p* < 0.001). There was a moderately positive correlation between mean scores of antibiotic knowledge and antibiotic use (r (359) = 0.55, *p* < 0.001) (Figure 6A). Also, a moderately positive correlation was found between the participants’ mean scores of knowledge on antibiotic resistance and antibiotic use scores (r (59) = 0.41, *p* < 0.001) (Figure 6B).

### 3.5. Duration of Antibiotic Use and Storage

The vast majority (41%) of the participants stated that they stored antibiotics in the barn/farm. The highest duration of antibiotic use among the farmers was three days with approximately 66%. The rate of residual antibiotic use for treatment of other sick animals was 67%. Interestingly more than 90% of the respondents expressed that they did not receive any training about antibiotic use, residues, and resistance (Table 2).

## 4. Discussion

Many factors play a role in the increasing development of antibiotic resistance, thus threatening public health by becoming a national and global problem. Prophylactic and therapeutic antibiotics commonly used in animals draw attention as one of the most important factors. Knowledge, attitudes, and behaviors of livestock farmers regarding the use of antibiotics in animals play a significant role in the development of antibiotic resistance. However, previous reports indicated that a significant portion of the farming community do not have sufficient information about antibiotic knowledge, antibiotic use, and resistance [5,14,16,17,18]. According to WHO reports on antibiotic resistance collected in 12 different countries, more than three out of four respondents in countries such as Sudan, Egypt, and India believed that viral infections of people such as colds and influenza could be treated with antibiotics. In a recent survey conducted in ruminant farmers by Sadiq et al., [16] more than 70% of respondents stated that all sick animals should be given antimicrobial agents, and 63% thought that antibiotics had no side effects in animals. In the current study, it was found that the proportion of respondents giving the same answers to similar questions was relatively low. On the other hand, 32% of the participants stated that antibiotics were effective against internal and external parasites in animals, and 61% considered antibiotics as antipyretic and analgesic. These findings indicate that the actual appropriate use of antibiotics is largely unknown. Antibiotics are among the chemicals used as growth factors in animals. Although until recently (for example see: https://www.fda.gov/animal-veterinary/development-approval-process/fact-sheet-veterinary-feed-directive-final-rule-and-next-steps) there was no legal obstacle toward the use of antibiotics in animals as a growth factor in the US, the use of antibiotics in animals for this purpose was prohibited in 2016 in the European Union countries. Within the framework of the harmonization laws of the European Union, Turkey started to apply this ban on the same date. For this reason, antibiotics used as growth factors are not produced or imported in the country. However, it is interesting to note that some farmers (41%) participating in this study believed that antibiotics can be used to improve weight gain in animals. This finding indicated that although antibiotics for growth promotion are not marketed, other antibiotics are likely to be used for this purpose. It is therefore necessary for the authorized institutions to apply stricter rules in order to prevent the use of antibiotics for growth promotion.

In this study, in addition to the knowledge on antibiotic use, the participants were observed to have insufficient awareness in terms of rational use of antibiotics. Approximately half of the respondents stated that when their animals became ill they used antibiotics they had readily available before they contacted the veterinarian. In a study conducted by Sudershan et al., [17] the rates of antibiotic use without consulting a veterinarian have been reported as 38% and 87% in urban and rural farmers respectively, which support our findings. Although there are many reasons that direct farmers to use antibiotics without consulting a veterinarian, self-diagnosis of diseases is a significant one. In a study on antibiotic use in goat farms conducted by Landfried et al., [15] the vast majority of farmers stated that they had sufficient knowledge about the behavior of animals and ability to identify abnormalities that might indicate a disease. The cost of veterinary services is one of the most important factors leading farmers to use antibiotics without consulting a veterinarian. A study from India showed that only one third of farmers apply to a veterinarian to reduce veterinary costs [14]. On the other hand, in many countries, farmers can easily buy antibiotics without the need for a prescription [14,19]. Although a number of measures have recently been taken to limit antibiotic use in human medicine in order to combat antibiotic resistance in Turkey, the implementation of these measures to the animal health field is progressing rather slowly and insufficiently. For this reason, animal owners can still easily access the antibiotics they want from veterinary clinics without any prescription. In the present study, a significant number of the participants preferred taking the recommendations of other farmers for the treatment of animal diseases with antibiotics. On the other hand, 84% of the participants took the advice of the veterinarian before using antibiotics. These data showed that farmers really valued advice from veterinarians, while they also benefited greatly from the experience of other farmers. Most of the participants were living in rural areas and it is well known in Turkey that rural farmers have difficulty to access to veterinary services when needed. Some regions covered in this study do not even provide adequate veterinary services due to the lack of sufficient veterinarians. For this reason, it is thought that farmers in rural areas need to exchange ideas with each other when treating sick animals.

As a matter of fact, the practices in dairy farms in India show that knowledge and beliefs are transferred from one generation to another [14]. Chauhan et al. [14] reported that the lack of official veterinary services in the community, easy access to antibiotics, and the need to provide profits and minimize losses caused an increase in non-prescription antibiotic consumption. The finding that one third of the participant used residual antibiotics for the treatment of animals can be the result of the above-mentioned concerns. The data of the present and previous studies suggest that there is an efficient socio-economic basis which encourages irrational antibiotic use by farmers both in Turkey and elsewhere. It is also a well-known fact that this situation is encouraged by those veterinarians who are more influenced by social expectations than scientific evidence [14].

After antibiotic resistance has reached serious levels, many organizations and countries, especially WHO, have spent great efforts to develop strategies for the prevention of resistance and raising awareness. For this purpose, many activities such as posters, informational advertisements, and meetings have been organized to educate the public and health workers. In fact, studies showed that healthcare workers were quite aware of the resistance problem [20,21,22]. However, it has been reported that farmers working in the livestock sector do not have sufficient awareness of the severity of the problems caused by antibiotic resistance [15,23]. In this study, the participants were firstly asked whether they had knowledge on antibiotic resistance before additional questions concerning antibiotic resistance and most of them responded negatively to this first question. It is not realistic to collect data about rational antibiotic use from farmers who are not aware of resistance. In fact, the basic condition for the resistance problem to be fully understood by society is to be trained on this subject. However, 91% of the respondents did not receive any training to acquire knowledge on antibiotics, their use, residues, and resistance. The participants who had knowledge about antibiotic resistance were asked the questions listed in Figure 5 and, most of them agreed that inappropriate use of antibiotics might lead to the development of resistant bacteria, resistance affects public health indirectly, and the frequent use of antibiotics in animals reduces their effects in the future. According to WHO reports, the proportion of those who thought antibiotic resistance was the biggest problem has been reported to be over 70% in many countries [5]. According to the results reported by Carter et al., [13] although people were aware that antibiotic misuse was associated with antibiotic resistance, they did not consider it as an important problem. Sadiq et al. [16] reported that the majority of farmers were not concerned about the effects of antimicrobial resistance on animal and public health. These data showed that the problems caused by antibiotic resistance were not completely understood by the farming community and as a result of which they were quite unaware of the severity of this issue.

One of the factors that facilitates antibiotic resistance is the use of antibiotics below the treatment dose and the failure to comply with the duration of treatment. It has been demonstrated by previous studies that people in the community have stopped and adjusted the antibiotic dose on their own initiative [24]. In our study, no question was asked to the participants about low-dose antibiotic use. However, as high as 45% of the respondents stated that they increased the dose of antibiotics and frequency of administration unless animals showed any signs of recovery. This finding indicated that the farmers took initiative in terms of adjusting antibiotic doses. In addition, 59% of the participants stated that they stopped using medication if animals were observed to be better one day after using antibiotics which revealed that appropriate duration of the treatment was not respected either. It is well known that all these misapplications may lead to an increase in antibiotic resistant bacteria.

A significant relationship between knowledge of antibiotic use and the level of education and other demographic factors such as the region of residence has been shown in previous studies [25,26,27]. On the other hand, the degree of this relationship varied from one country to another in terms of gender and age. While most of the studies reported a correlation between antibiotic knowledge and age and sex factors [28,29], some studies failed to find a statistically significant association between these parameters [30]. In the present study, a statistically significant association was found between antibiotic knowledge, use, and resistance and demographic parameters such as age and location. In Turkey, people dealing with animal husbandry generally live in rural areas, have low education levels, and are relatively old people. Livestock production is generally considered as a necessity rather than as a professional field. The findings of this study substantiated this as most of the farmers participating in the study were primary education graduates and 43% of them were at the age of 48 years and more. In contrast to the findings of the present study, studies carried out in other countries reported that most of the farmers were younger and well educated [16]. It is therefore believed that encouraging young people with a high level of education to get involved in the livestock sector will increase the perception and awareness of antibiotic resistance.

## 5. Conclusions

In conclusion, this survey revealed viewpoints of cattle breeders located in eastern Turkey on antibiotic knowledge, use, and resistance which may help the authorities to draw a direction toward taking necessary steps in order to minimize antibiotic resistance. Periodic educational programs are urgently needed to train farmers in order to raise awareness about antibiotics, rational drug use, and resistance with particular attention to public health as well as alternative approaches such as vaccination, environmental protection, and preventive medicine. In addition, necessary measures should be taken by the authorities to limit and control the use of veterinary drugs. It is suggested that many aspects of the problem of antibiotic resistance can be overcome by identifying and communicating effectively with all stakeholders. In addition, there may be a need for some surveillance of antibiotic use and antimicrobial resistance.

## Figures and Tables

**Table 1:** Demographic data of farmers participating in the survey.

**Gender**							
	Female				Male		
N = 360	6				354		
%	1.66				98.33		
**Age Range (Year)**							
	18–22	23–27	28–32	33–37	38–42	43–47	48 or more
N = 360	7	15	38	32	49	63	156
%	1.94	4.16	10.55	8.88	13.61	17.5	43.33
**Educational Status**							
	Uneducated	Primary School	High School	Associate Degree	Bachelor Degree		Postgraduate
N = 360	50	223	48	19	14		6
%	13.88	61.94	13.33	5.27	3.88		1.66
**Residence**							
	Urban				Rural		
N = 360	74				286		
%	20.55				79.44		
**The Time Spent as Farmer (Years)**							
	0–3		3–6		6–10		10 or more
N = 360	20		20		54		266
%	5.55		5.55		15		73.88

**Table 2:** The data obtained from the participants about duration of antibiotic use and storage.

**Where do You Store the Antibiotics?**								
	In the barn	In any part of house		Medicine cabinet		Refrigerator		Other
N = 360	146	24		76		110		4
%	40.6	6.7		21.1		30.6		1.1
**How Many Days do You Use Antibiotics to Cure Sick Animals? (Days)**								
	1	2	3	4	5	6	7	7 or more
N = 360	9	18	239	37	28	3	22	4
%	2.5	5.0	66.4	10.3	7.8	0.8	6.1	1.1
**Would You Use the Residual Antibiotics for the Treatment of Other Sick Animals Later on?**								
	Yes				No			
N = 360	240				120			
%	66.7				33.3			
**How Long do You Store the Residual Antibiotics For reuse? (Months)**								
	I don’t store	1		3	6	9	12	12 or more
N = 360	101	102		68	15	1	22	51
%	28.1	28.3		18.9	4.2	0.3	6.1	14.2
**How Frequently do You Give Antibiotics to Animals?**								
	Every week	Every month		Every three months	Every six months	Once a year		Only when the animal is sick
N = 360	8	16		9	11	3		313
%	2.2	4.4		2.5	3.1	0.8		86.9
**Did You Receive Any Training on These Listed Subjects?**								
	Antibiotic use in animals	Antibiotic resistance		Antibiotic residues in foods	General information about antibiotics	I haven’t received any training at all		I received training on all subjects
N=360	12	5		1	8	328		6
%	3.3	1.4		0.3	2.2	91.1		1.7
